# The eye as a gateway to the brain: mechanisms of ocular neuroinvasion by parasitic pathogens

**DOI:** 10.3205/dgkh000668

**Published:** 2026-07-28

**Authors:** Sona Bhardwaj, Sweety Kumari, Rijhul Lahariya, Mainak Sinha, Anand Kumar Das, Simmi Kishore, Gargee Anand, Saraj Kumar Singh

**Affiliations:** 1 Department of Microbiology, ESIC Hospital, Patna, Bihar, India; 2 Department of Ophthalmology, MediCiti Institute of Medical Sciences, Hyderabad, Telangana, India; 3 All India Institute of Medical Sciences, Patna, Bihar, India; 4 Department of Neurosurgery, All India Institute of Medical Sciences, Patna, Bihar, India; 5 Department of Anaesthesiology and Critical Care Medicine, Indira Gandhi Institute of Medical Sciences, Patna, Bihar, India; 6 Department of Microbiology, All India Institute of Medical Sciences, Patna, Bihar, India

**Keywords:** ocular neuroinvasion, eye-brain axis, parasitic infections, central nervous system dissemination, blood-retinal barrier

## Abstract

The central nervous system (CNS) is protected by specialized anatomical and immunological barriers that limit pathogen entry; however, several infectious agents have evolved mechanisms to circumvent these defenses. While hematogenous dissemination and peripheral nerve invasion are well-recognized routes of neuroinfection, the eye has received comparatively little attention as a potential gateway to the brain. Owing to its embryological origin from the neuroectoderm, direct anatomical continuity with the CNS through the optic nerve, specialized blood–retinal barrier, and immune-privileged environment, the eye represents a unique neuroimmune interface that may facilitate pathogen persistence and dissemination.

Parasitic pathogens, including *Toxoplasma (T.) gondii*, *Acanthamoeba* spp., *Toxocara* spp., *Taenia (T.) solium*, and *Loa (L.) loa*, can establish ocular infection and, under certain conditions, contribute to neurological involvement.

This review examines the anatomical and immunological features that make the eye a potential neuroinvasive niche and synthesizes current evidence supporting ocular-to-CNS dissemination. Major mechanisms include infected immune-cell trafficking (“Trojan horse” transport), inflammation-induced disruption of the blood–retinal barrier, local neuroinflammatory responses, optic nerve–associated spread, and persistence within ocular reservoirs followed by reactivation. Among currently studied parasites, *T. gondii* provides the strongest evidence for biologically plausible ocular neuroinvasion, whereas helminths and free-living amoebae highlight additional pathways linking ocular and neurological disease. Although direct proof of ocular-to-brain dissemination remains limited for many pathogens, accumulating experimental and clinical observations support the concept that ocular infection may have broader neurological implications. Understanding the eye–brain interface may improve recognition of neuroinvasive disease and reveal novel targets for therapeutic intervention and prevention.

## Abstract (de)

Das zentrale Nervensystem (ZNS) wird durch hochspezialisierte anatomische und immunologische Barrieren geschützt, die das Eindringen von Krankheitserregern begrenzen. Dennoch haben zahlreiche Infektionserreger Mechanismen entwickelt, um diese Schutzsysteme zu überwinden. Während die hämatogene Ausbreitung und die Invasion über periphere Nerven als etablierte Wege der Neuroinvasion gelten, wurde das Auge als potenzielles Tor zum Gehirn bislang vergleichsweise wenig untersucht. Aufgrund seines neuroektodermalen Ursprungs, der direkten anatomischen Verbindung zum ZNS über den Sehnerv, der spezialisierten Blut-Retina-Schranke sowie seines immunprivilegierten Milieus stellt das Auge eine einzigartige neuroimmunologische Schnittstelle dar, die die Persistenz und Ausbreitung von Pathogenen begünstigen kann.

Parasitäre Erreger wie *Toxoplasma (T.) gondii*, Acanthamoeba-Arten, Toxocara-Arten, *T. solium* und* L. loa* können okuläre Infektionen verursachen und unter bestimmten Bedingungen zu neurologischen Manifestationen beitragen.

Der Übersichtsartikel beleuchtet die anatomischen und immunologischen Eigenschaften, die das Auge zu einer potenziellen neuroinvasiven Nische machen, und fasst die derzeitige Evidenz für eine Ausbreitung vom Auge zum ZNS zusammen. Zu den wichtigsten Mechanismen zählen der Transport infizierter Immunzellen („Trojanisches-Pferd“-Mechanismus), die entzündungsbedingte Störung der Blut-Retina-Schranke, lokale neuroinflammatorische Prozesse, eine mögliche Ausbreitung entlang des Sehnervs sowie die Persistenz von Parasiten in okulären Reservoiren mit späterer Reaktivierung. Unter den bislang untersuchten Parasiten liefert *T. gondii* die überzeugendsten Hinweise auf eine biologisch plausible okuläre Neuroinvasion, während Helminthen und freilebende Amöben weitere Verbindungen zwischen okulären und neurologischen Erkrankungen aufzeigen. Obwohl für viele Erreger ein direkter okulär-zerebraler Ausbreitungsweg noch nicht eindeutig nachgewiesen wurde, sprechen zunehmende experimentelle und klinische Befunde dafür, dass okuläre Infektionen relevante neurologische Auswirkungen haben können. Ein besseres Verständnis der Augen-Gehirn-Schnittstelle könnte die frühzeitige Erkennung neuroinvasiver Erkrankungen verbessern und neue Ansatzpunkte für Prävention und Therapie eröffnen.

## Introduction

The central nervous system (CNS) is protected by a series of highly specialized anatomical and immunological barriers that restrict pathogen entry and preserve neural homeostasis as neurovascular unit [1]. Despite these defenses, numerous infectious agents have evolved strategies to invade neural tissues, resulting in significant neurological morbidity and mortality worldwide [2], [3], [4]. While hematogenous dissemination and peripheral nerve invasion are widely recognized routes of neuroinvasion, the potential contribution of the eye as a gateway to the brain has received comparatively less attention [5].

The eye occupies a unique position among peripheral organs because of its intimate developmental, anatomical, and functional relationship with the CNS [6]. During embryogenesis, the retina and optic nerve arise directly from the neuroectoderm of the developing forebrain, rendering the posterior segment of the eye an extension of neural tissue rather than a conventional sensory organ [7]. This continuity is further reinforced by the optic nerve, retinal ganglion cell axons, shared neurovascular characteristics, and similarities between the blood–retinal barrier and the blood–brain barrier. Consequently, pathological processes affecting ocular tissues may have implications that extend beyond visual dysfunction and potentially influence CNS health.

In addition to its neuroanatomical connections, the eye possesses a specialized immune environment characterized by immune privilege [8]. This state limits excessive inflammatory responses that could compromise vision but may simultaneously facilitate pathogen persistence [9], [10]. Several parasitic organisms are capable of establishing prolonged residence within ocular tissues, where reduced immune surveillance and restricted immune effector activity can provide a protected niche for survival [11]. Persistent ocular infection may therefore create opportunities for dissemination through local neural pathways, infected immune cells, or inflammation-mediated barrier disruption.

Parasitic infections represent an important but underexplored component of ocular neuroinvasion. Protozoan parasites such as *Toxoplasma (T.) gondii*, migratory helminths including *Toxocara* spp. and *T. solium*, and free-living amoebae such as *Acanthamoeba* spp. have all been associated with ocular disease and, under certain circumstances, CNS involvement [12]. Although direct evidence supporting ocular-to-brain dissemination varies considerably among pathogens, accumulating experimental, pathological, and clinical observations suggest that ocular tissues may function not only as sites of infection but also as potential reservoirs or intermediate platforms facilitating neuroinvasive processes.

Understanding how parasites exploit the eye–brain interface is important for several reasons. First, it may provide insights into previously underappreciated mechanisms of CNS invasion. Second, recognition of ocular involvement may improve early diagnosis of neuroinvasive disease. Finally, elucidating the biological pathways linking ocular infection to neurological pathology may identify novel targets for therapeutic intervention and disease prevention. This review examines the anatomical and immunological features that make the eye a potential neuroinvasive niche, explores the major mechanisms by which parasitic pathogens may disseminate from ocular tissues to the CNS, and synthesizes current evidence from major ocular parasitic infections to evaluate the role of the eye as a gateway to the brain.

## Method

A narrative literature review was conducted to evaluate current evidence regarding the role of the eye as a potential gateway for CNS invasion by parasitic pathogens. Relevant literature was identified through searches of PubMed and Google Scholar up to June 2026. Search terms included combinations of “ocular parasitosis,” “ocular toxoplasmosis,” “ocular neuroinvasion,” “eye–brain axis,” “optic nerve dissemination,” “blood–retinal barrier,” “ocular immune privilege,” “neurocysticercosis,” “toxocara,” “acanthamoeba,” and “parasite dissemination.”

Original research articles, experimental animal studies, pathological investigations, clinical reports, and review articles addressing ocular infection, neuroinvasion mechanisms, blood–retinal barrier dysfunction, optic nerve involvement, immune privilege, and CNS manifestations of parasitic diseases were considered. Articles focused exclusively on non-parasitic infections or unrelated ocular diseases were excluded. Additional studies were identified through manual screening of reference lists from relevant publications.

The retrieved literature was synthesized thematically into four major domains:

1. anatomical and immunological characteristics of the eye–brain interface,
2. mechanisms of ocular-to-CNS dissemination,
3. evidence from major parasitic infections with ocular and neurological involvement, and
4. current limitations and future research directions.

## Results

### The eye–brain interface: why the eye represents a potential neuroinvasive portal

The eye possesses several unique structural and immunological characteristics that distinguish it from most peripheral organs and make it a potentially important interface between the external environment and the CNS [9]. These features provide a biological framework through which pathogens, including parasites, may establish local infection, evade immune elimination, and potentially gain access to neural tissues.

Unlike most sensory organs, the retina originates directly from the neuroectoderm of the developing forebrain and remains anatomically connected to the CNS throughout life. The optic nerve is composed of retinal ganglion cell axons that project directly to intracranial visual centers and is enveloped by meningeal layers that are continuous with those surrounding the brain [13]. Consequently, the posterior segment of the eye represents specialized neural tissue rather than a conventional peripheral organ [14].

This close anatomical relationship has important implications for infectious diseases. Pathogens capable of surviving within retinal cells, glial cells, or tissues adjacent to the optic nerve may encounter neural structures that provide potential routes for local dissemination [15]. Although the relative contribution of optic nerve–associated spread remains incompletely defined for many parasitic infections, the existence of direct neural continuity supports the concept that ocular infection can have neurological consequences beyond visual impairment.

Preservation of vision requires strict regulation of inflammation within ocular tissues. As a result, the eye maintains a state of immune privilege characterized by reduced expression of inflammatory mediators, local production of immunosuppressive factors, and mechanisms that limit immune-mediated tissue damage [16]. While these adaptations protect delicate visual structures, they may also create conditions favorable for prolonged pathogen survival.

Many parasites have evolved sophisticated strategies to exploit immunologically protected environments. Within the eye, reduced immune surveillance may facilitate persistence of intracellular parasites, chronic tissue colonization, or establishment of latent stages that can remain viable for extended periods [17]. Such long-term reservoirs may increase opportunities for subsequent dissemination, particularly when local inflammation, immune dysregulation, or systemic immunosuppression occurs.

The blood–retinal barrier (BRB) serves as the ocular counterpart of the blood–brain barrier (BBB), regulating molecular and cellular trafficking between the circulation and retinal tissues. Tight junctions between endothelial and retinal pigment epithelial cells restrict pathogen entry while maintaining retinal homeostasis [18]. However, infection-induced inflammation can compromise barrier integrity, increase vascular permeability and promote infiltration of immune cells. Barrier disruption is particularly relevant in parasitic infections because many neuroinvasive pathogens exploit inflammatory responses to facilitate dissemination [19]. Infected leukocytes, parasite-derived molecules, and host inflammatory mediators may collectively alter BRB function, creating opportunities for movement of pathogens into retinal tissues or from ocular sites toward adjacent neural structures. Thus, the eye–brain interface should be viewed not merely as a passive barrier system but as a dynamic neuroimmune environment in which host defenses and pathogen survival strategies continuously interact.

### Mechanistic pathways of ocular-to-CNS dissemination

Once parasites establish infection within ocular tissues, several biological mechanisms may facilitate their movement toward the CNS. Although the relative importance of each pathway differs among parasites, most neuroinvasive processes involve a combination of pathogen survival, host immune responses, and disruption of normal tissue barriers.

One of the best-described mechanisms of parasite dissemination is the "Trojan horse" strategy [5]. In this process, parasites infect host immune cells such as monocytes, macrophages, or dendritic cells and use them as vehicles for transport throughout the body [20]. Rather than moving independently, the parasite remains hidden within migrating cells and is carried across biological barriers.

This mechanism has been studied most extensively in *Toxoplasma gondii* [21]. After infection, the parasite can invade circulating immune cells and alter their migratory behavior, allowing infected cells to travel through blood vessels and enter distant tissues [22]. Because immune cells naturally patrol both ocular and neural environments, they may serve as important links between local ocular infection and CNS dissemination. Beyond simple transport, infected immune cells can release inflammatory mediators that increase vascular permeability and promote further pathogen spread [23]. As a result, the Trojan-horse mechanism not only facilitates parasite movement but may also create conditions that favor subsequent tissue invasion.

The optic nerve provides a direct anatomical connection between the eye and the brain. Because retinal ganglion cell axons extend from the retina into intracranial visual pathways, infection involving retinal tissues may place parasites in close proximity to neural structures. Several experimental studies have suggested that pathogens may spread along optic nerve-associated tissues following ocular infection [24]. Inflammatory changes involving the optic nerve have been observed in a variety of ocular parasitic diseases, supporting the possibility of local neural dissemination [24]. However, direct evidence demonstrating active parasite migration through the optic nerve remains limited for most organisms. Despite these uncertainties, the optic nerve remains an attractive biological route because it bypasses many of the challenges associated with systemic dissemination and provides immediate access to CNS structures.

The blood–retinal barrier normally restricts movement of pathogens and immune cells into retinal tissues. During parasitic infection, however, inflammatory responses may compromise barrier integrity. Cytokines, chemokines, and other inflammatory mediators released by both host cells and parasites can weaken tight junctions and increase vascular permeability [25], [26]. Barrier disruption may allow infected immune cells, parasite-derived products, or free parasites to move more easily between the circulation and ocular tissues [27]. In addition, inflammation can extend beyond the site of infection, creating a microenvironment that supports dissemination toward neighbouring neural structures. Importantly, neuroinflammation is not simply a consequence of infection but may actively contribute to disease progression [28]. Excessive inflammatory responses can damage protective barriers, alter neuronal function, and facilitate pathogen access to previously protected compartments.

**Figure 1:**
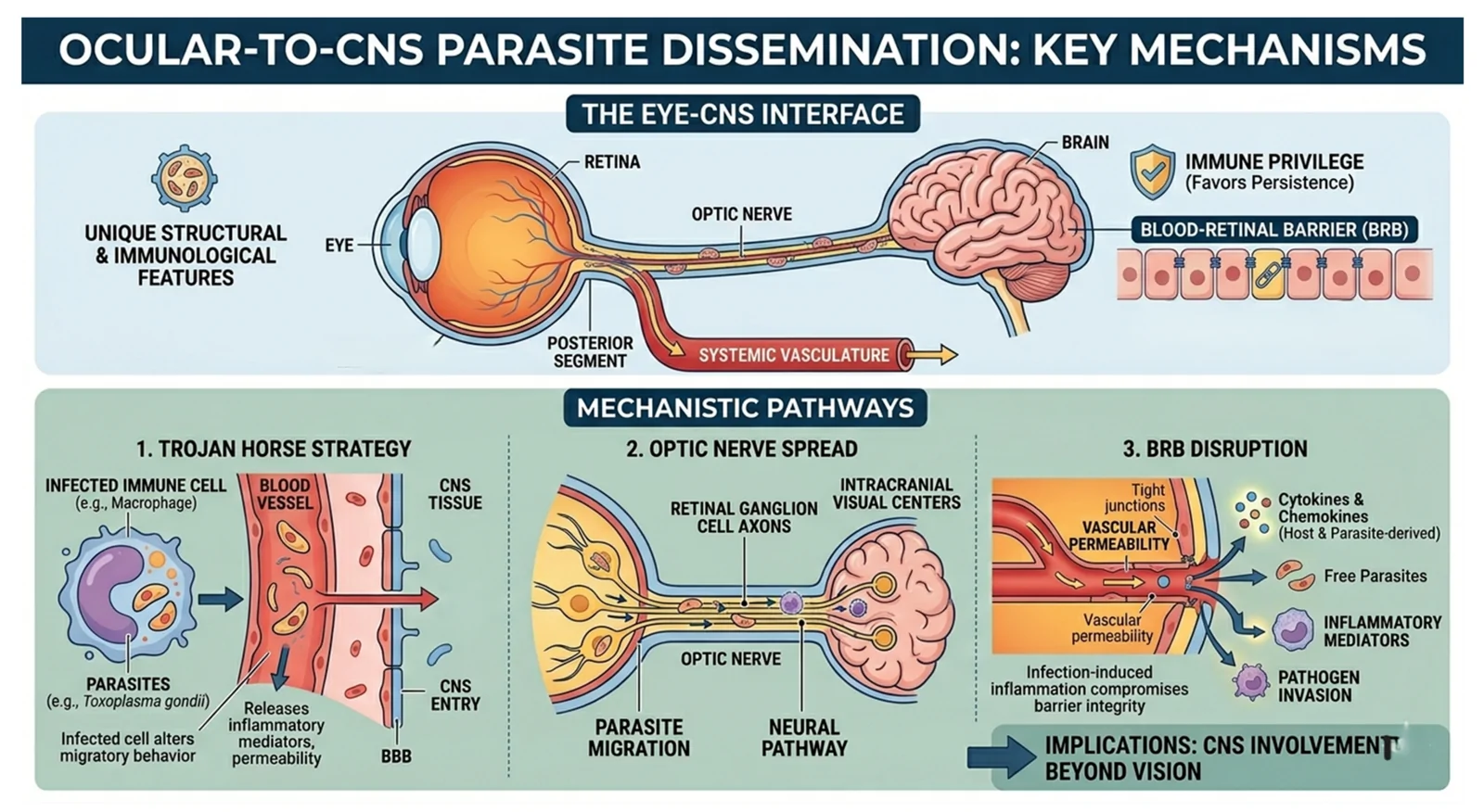
Key mechanisms showing how a parasite can disseminate to CNS through ocular infection.

Many parasitic infections are characterized by the ability to persist within host tissues for prolonged periods. The immune-privileged nature of the eye may support the formation of long-term reservoirs where parasites remain viable despite host immune responses. Persistent infection is particularly important because dissemination does not always occur during the initial stage of disease [29]. In some cases, parasites may remain dormant or clinically silent for months or years before reactivation. Local inflammation, immune suppression, or changes in host immunity may then trigger renewed parasite replication and increase the likelihood of spread beyond ocular tissues. This concept is especially relevant for chronic ocular toxoplasmosis, in which recurrent episodes of retinal inflammation often arise from persistent tissue cysts. Similar principles may apply to other parasitic infections capable of prolonged survival within ocular environments (Figure 1 (Fig. 1)).

### Major parasitic infections supporting ocular-to-CNS dissemination

Evidence for ocular neuroinvasion varies considerably among parasitic pathogens. While some organisms have well-established ocular and neurological manifestations, others are supported primarily by experimental or limited clinical observations. Despite these differences, many parasites share common themes, including persistence within ocular tissues, induction of local inflammation, and exploitation of host cellular or neural pathways. Representative examples are summarized below (Table 1 (Tab. 1)).

**Figure 2:**
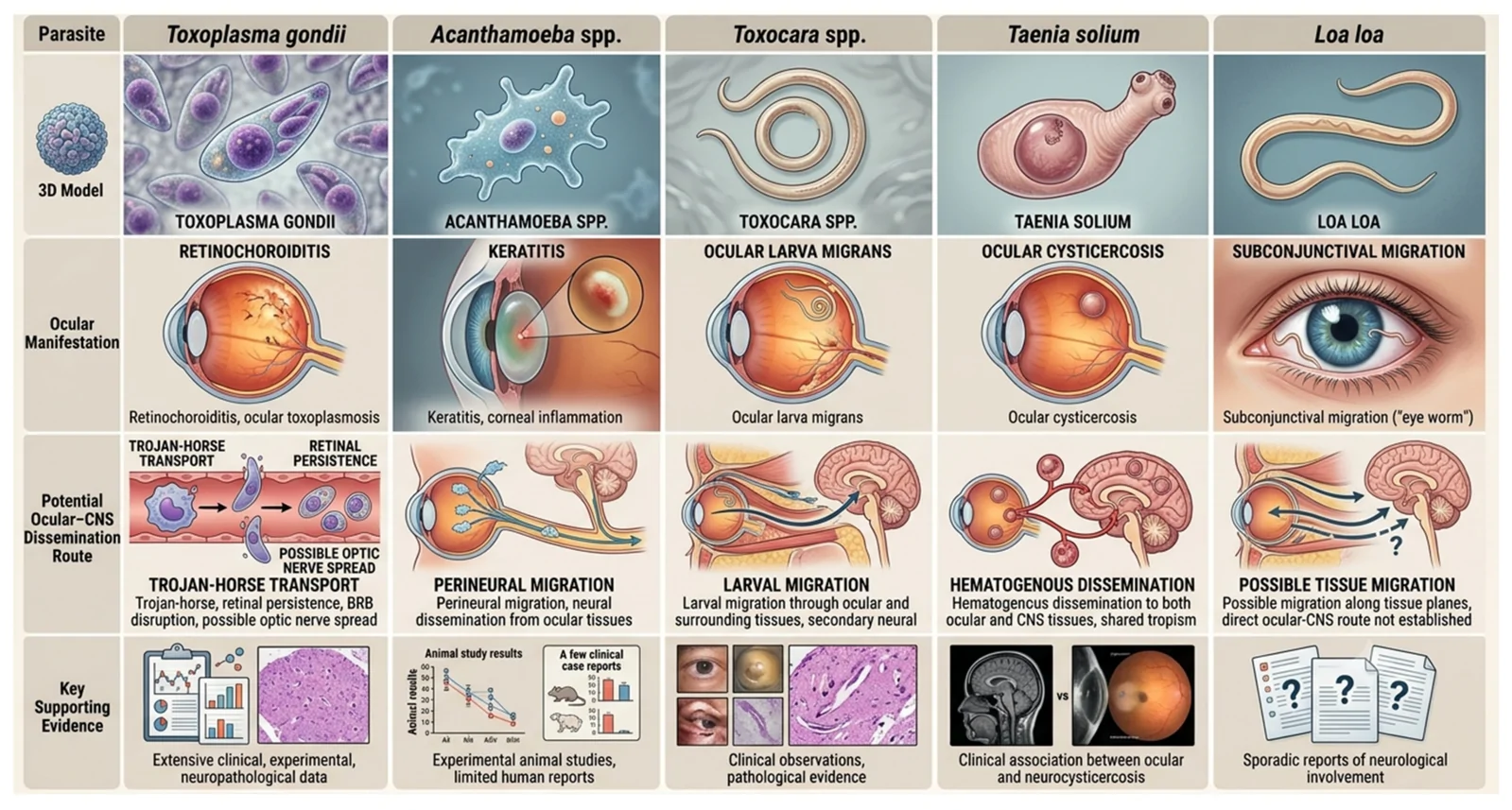
Various parasites, their ocular manifestations, their route of dissemination have been summarised.

Among these pathogens, *Toxoplasma gondii* provides the strongest evidence supporting a biologically plausible connection between ocular infection and CNS dissemination [30]. In contrast, helminth infections more commonly demonstrate shared ocular and neurological involvement resulting from tissue migration rather than proven direct ocular-to-brain spread [31]. Free-living amoebae such as *Acanthamoeba* spp. occupy an intermediate position, with experimental findings suggesting neural dissemination but relatively limited human evidence [32], [33]. Collectively, these observations indicate that ocular infection should not always be viewed as an isolated disease process. Instead, the eye may serve as a site of persistence, a source of inflammatory signaling, or a potential staging point from which parasites interact with neural tissues and, under appropriate conditions, contribute to CNS involvement (Figure 2 (Fig. 2)).

## Discussion

The present review highlights the eye as a biologically plausible but underrecognized interface for parasitic neuroinvasion. Traditionally, CNS invasion by pathogens has been viewed primarily through the lens of hematogenous dissemination or peripheral nerve invasion. However, the unique developmental and anatomical relationship between the eye and the brain suggests that ocular tissues may play a more active role in neuroinvasive processes than is commonly appreciated. The retina and optic nerve are direct extensions of the CNS, while the blood–retinal barrier and ocular immune environment share several functional similarities with protective systems of the brain. Collectively, these features create conditions in which parasites may establish local infection, evade immune clearance, and potentially interact with neural structures.

Among the parasitic pathogens reviewed, *T. gondii* provides the strongest evidence supporting a potential ocular contribution to CNS dissemination. Its ability to infect migratory immune cells, persist within retinal tissues, and induce inflammatory alterations of barrier systems offers a mechanistic framework linking ocular infection with neurological involvement. In contrast, evidence for helminthic infections such as Toxocara spp. and *T. solium* is less direct and largely reflects shared tissue tropism and migratory behavior rather than proven ocular-to-brain transmission. Similarly, although experimental studies have demonstrated neural dissemination by Acanthamoeba spp., direct clinical evidence supporting ocular-origin CNS invasion remains limited. These observations suggest that the likelihood and importance of ocular neuroinvasion may vary substantially among different parasitic organisms.

A recurring theme emerging from the literature is the dual role of ocular immune privilege. While immune privilege is essential for preserving vision by limiting excessive inflammatory damage, it may simultaneously create a favorable niche for pathogen persistence. Chronic survival of parasites within ocular tissues increases the possibility of prolonged host–pathogen interactions, recurrent inflammatory episodes, and opportunities for dissemination beyond the eye. This concept is particularly relevant for pathogens capable of establishing latent or persistent stages, where ocular tissues may function as reservoirs rather than merely sites of acute disease.

Despite increasing recognition of the eye–brain interface in infectious diseases, direct evidence supporting ocular-to-CNS dissemination remains limited for most parasitic pathogens. Much of the current understanding is derived from experimental models, isolated case reports, or indirect pathological observations. The relative contribution of ocular routes compared with conventional hematogenous dissemination is still unclear, and definitive proof of parasite migration through optic nerve-associated pathways is lacking for many organisms. Future studies integrating advanced imaging, molecular tracing techniques, and longitudinal clinical data are needed to clarify the mechanisms, frequency, and clinical significance of ocular neuroinvasion in parasitic infections.

## Conclusion

The eye represents a unique anatomical and immunological interface with the central nervous system, combining neural continuity, immune privilege, and specialized barrier systems that may create opportunities for pathogen persistence and dissemination. Although the strength of evidence varies among parasitic infections, available experimental and clinical data suggest that ocular tissues can function as more than isolated sites of disease. Through mechanisms such as infected immune-cell trafficking, barrier disruption, local neuroinflammation, and potential neural spread, parasites may exploit the eye–brain axis to facilitate CNS involvement. Among currently studied pathogens, *T. gondii* provides the most compelling model of ocular neuroinvasion, while evidence from helminthic infections and free-living amoebae highlights additional routes by which ocular and neurological disease may be linked. Collectively, these findings support a broader view of ocular parasitic infections as conditions with potential neurological implications. Improved understanding of the eye–brain interface may enhance recognition of neuroinvasive disease and provide new insights into host–parasite interactions at one of the body's most specialized biological boundaries.

## Notes

### Authors’ contributions

Sona Bhardwaj and Sweety Kumari contributed equally.

### Authors’ ORCIDs

- Lahariya R: https://orcid.org/0009-0003-5769-4509
- Sinha M: https://orcid.org/0000-0002-2286-0701
- Das AK: https://orcid.org/0000-0002-0705-9393
- Singh SK: https://orcid.org/0000-0003-3156-7096
- Anand G: https://orcid.org/0009-0008-0473-389X

### Funding

None.

### AI usage

AI was used solely for the generation of graphical illustrations in this manuscript using Google Gemini from prompts independently provided by the authors. All scientific information and intellectual contributions originated from and were reviewed by the authors. 

### Competing interests

The authors declare that they have no competing interests.

## Figures and Tables

**Table 1:**
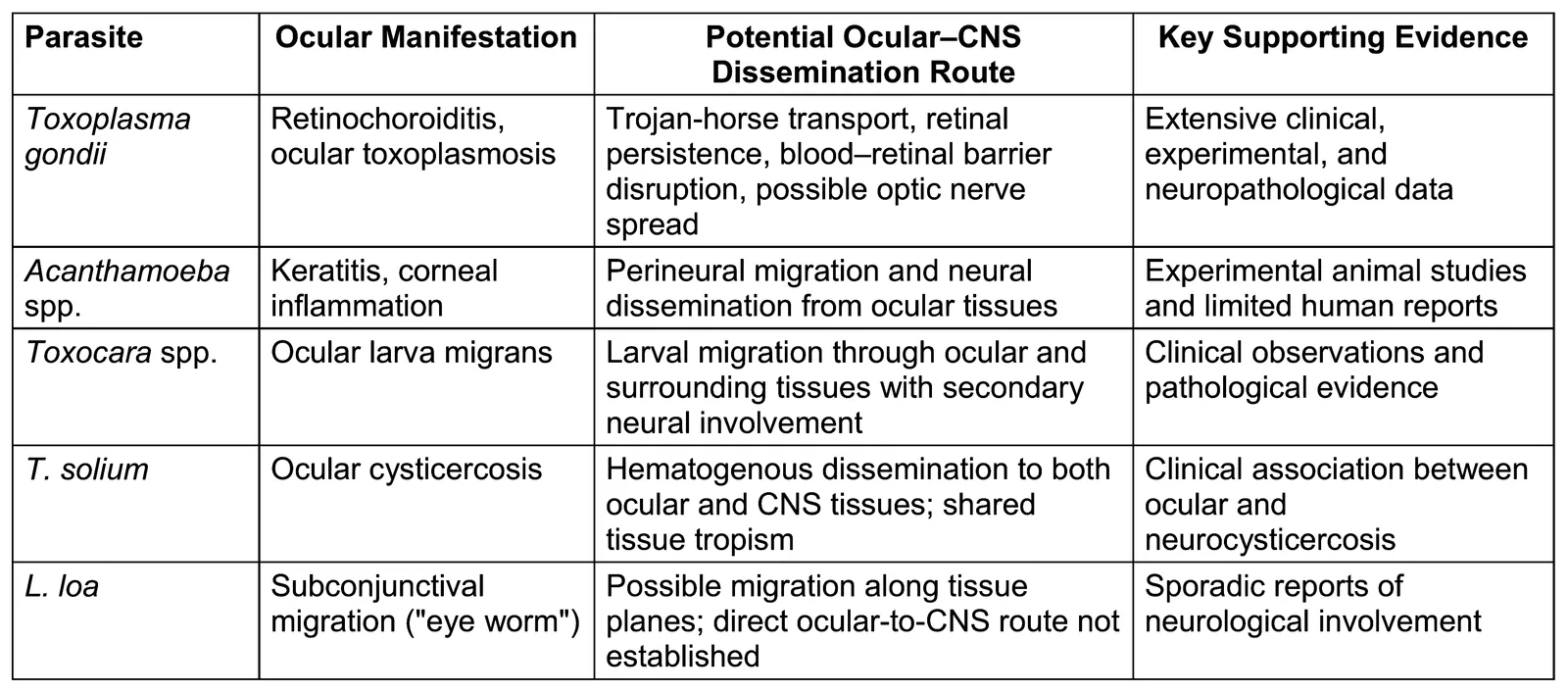
Representative parasitic pathogens with ocular involvement and proposed mechanisms linking ocular infection to CNS dissemination
